# Serum sodium and chloride are inversely associated with dyskinesia in Parkinson's disease patients

**DOI:** 10.1002/brb3.867

**Published:** 2017-11-09

**Authors:** Cheng‐jie Mao, Chong‐ke Zhong, Yong Yang, Ya‐ping Yang, Fen Wang, Jing Chen, Jin‐ru Zhang, Hui‐jun Zhang, Hong Jin, Ling‐li Xu, Juan‐ying Huang, Chun‐Feng Liu

**Affiliations:** ^1^ Department of Neurology and Suzhou Clinical Research Center of Neurological Disease the Second Affiliated Hospital of Soochow University Suzhou China; ^2^ Department of Epidemiology School of Public Health Medical College of Soochow University Suzhou China; ^3^ Clinical Laboratory The Second Affiliated Hospital of Soochow University Suzhou China; ^4^ Institute of Neuroscience Soochow University Suzhou China

**Keywords:** chloride, Dyskinesia, Parkinson's disease, Sodium

## Abstract

**Objective:**

We aim to report and evaluate the associations between serum sodium and chloride and dyskinesia in patients with Parkinson's disease. One hundred and two patients with Parkinson's disease were enrolled in this study.

**Methods:**

Patients’ serum electrolytes including sodium, calcium, potassium, magnesium, and chloride were measured. Other demographic information was collected, and Unified Parkinson's disease rating scale and Hoehn and Yahr stage scale were also performed.

**Results:**

Patients with dyskinesia tended to have longer duration of disease, higher daily levodopa‐equivalent dose, and Hoehn–Yahr stage, with lower serum sodium than those without dyskinesia. Spearman correlation analyses showed that serum sodium inversely correlated with duration of disease (*r* = −.218, *p *=* *.028), and positively correlated with serum chloride levels (*r* = .565, *p *<* *.001). Univariate logistic regression analysis found that duration of disease, daily levodopa‐equivalent dose, serum sodium, and serum chloride were associated with dyskinesia in Parkinson's disease patients (*p *<* *.05 for all). After adjusting for age, sex, age at onset of Parkinson's disease, medical history, and other covariates, serum sodium and chloride were still associated with dyskinesia, with corresponding Odd ratios 0.783 (95% confidence intervals, 0.642–0.955) and 0.796 (95% confidence intervals, 0.652–0.972), respectively.

**Conclusion:**

Our findings indicated that serum sodium and chloride levels were inversely associated with dyskinesia in patients with Parkinson's disease. Further studies with large samples and range of serum sodium and chloride are needed.

## 1 INTRODUCTION

Parkinson's disease (PD) is the second most common neurodegenerative disease, and is characterized by motor and nonmotor dysfunctions (Hattori, 2016). Although L‐dopa is the most effective drug for the symptomatic treatment of PD, the emergence of side effects, particularly motor fluctuations and dyskinesia can limits its use in some patients (Jankovic, 2005). Changes in cellular signaling pathways, and enhanced D1 stimulation causing widespread molecular adaptations in striatal medium spiny neurons play important roles in Levodopa‐induced dyskinesia (LID) (Hechtner et al., 2014). Other risk factors that have been identified related to dyskinesia in PD patients include a younger onset‐age, female sex, duration of levodopa therapy, disease duration, and the total daily levodopa dose (Hauser, McDermott, & Messing, 2006; Ku & Glass, 2010; Kumar, Van Gerpen, Bower, & Ahlskog, 2005; Manson, Stirpe, & Schrag, 2012).

Sodium is the major osmotically active solute in the extracellular compartment (Adrogue & Madias, 2000). Sodium concentration is regulated by numerous factors including ion channels, and hormones. The Na+‐K+ pump, and many ion channels are important for maintaining the osmotic and electrochemical gradients across cell membrane. The concentration of sodium is 11 millimolar (mm) both in gray and white matter in the brain, 10–14 mm within neuron cells, and 140 mm outside neuron cells. A constant gradient of concentrations across the membrane plays a very important role in calcium regulation homeostasis, cell volume control, glucose transport, the electric potential of membranes, pH regulation, and neurotransmission (Madelin, Kline, Walvick, & Regatte, 2014). Ion channels in the dopaminergic neurons of the substantia nigra including epithelial sodium channels, G‐protein‐coupled inwardly rectifying potassium channel (GIRK), calcium‐sensitive potassium channels, and N‐methyl‐D‐aspartate (NMDA) receptors may be associated with the development of dyskinesia (Dragicevic, Schiemann, & Liss, 2015).

Chloride is the body's main anion, representing 70 percent of the total negative ion content (Yunos, Bellomo, Story, & Kellum, 2010). Chloride is the most important extracellular anion and plays important roles in many body functions, including the maintenance of osmotic pressure, acid–base balance, muscular activity, and the movement of water between fluid compartments. Effective synaptic inhibition is crucial for proper neural coding. Fast inhibition in the central nervous system primarily relies on chloride moving into the postsynaptic neuron. Chloride dysregulation is implicated in some neurological and psychiatric disorders (Doyon, Vinay, Prescott, & De Koninck, 2016). Increased death of dopaminergic neurons due to hypochlorite‐dependent conversion to a cytotoxic redox‐ cycling product, leading to the generation of reactive oxygen species and oxidative stress, may be a factor in the pathogenesis of PD (Mehta et al., 2016).

To the best of our knowledge, there are no studies that correlate serum sodium and chloride with dyskinesia in PD patients. However, due to the important role of sodium and chloride in the central nervous system, we do believe the relationship exists between abnormal concentrations of serum sodium and chloride and dyskinesia. In this study, we aim to investigate the relationship between serum sodium and chloride and dyskinesia in PD patients.

## 2 METHODS

### 2.1 Subjects

This was a retrospective study in which 102 outpatient PD patients, who had been tested for fasting electrolytes, were enrolled at the Second Affiliated Hospital of Soochow University from Jan 2014 to Dec 2016. The clinical diagnosis of idiopathic PD was determined based on UK Parkinson's disease Brain Bank criteria (Hughes, Ben‐Shlomo, Daniel, & Lees, 1992). Demographic information, including age, sex, disease duration, concurrent diseases, combined medication, and detailed medical history, was collected. All the subjects were carefully evaluated by a movement disorder specialist. Unified Parkinson's disease rating scale (UPDRS) and Hoehn & Yahr stage (H&Y stage) scale were applied to all PD subjects (Fahn & Parkinson Study, 2005; Hoehn & Yahr, 1967). Dyskinesia was recorded depending on the part four of UPDRS.

Blood was obtained from the antecubital vein in all subjects between 8.30 and 10.00 a.m., Laboratory tests were done to evaluate the concentration of electrolytes including sodium, calcium, magnesium, potassium and chloride by an Olympus Au5400 automatic biochemical analyzer (First Chemical Co., LTD, Japan). In order to reduce the impacts of medications and foods, all PD patients completed the electrolyte detection in fasting state. PD patients who took the antiparkinsonism medications as usual after blood collection and completed the neurological assessments in “ON” state were included in the study.

Subjects were excluded if they had a secondary parkinsonism syndrome, atypical parkinsonian syndrome, malignant neoplasm, epilepsy, or severe cardiopulmonary disease. Forty‐one drug naïve patients were also excluded because dyskinesia is typically induced by the dopaminergic treatment. Four patients who were treated with diuretics for hypertension were also excluded from the study.

This study was approved by the ethics committee of the Second Affiliated Hospital of Soochow University and informed consent was obtained from all participants.

### 2.2 Statistics

The study participants were divided into two groups: with or without dyskinesia. Continuous variables were expressed as mean ± standard deviation (SD) or median (interquartile range [IQR]) and were compared using Student's *t* test or Wilcoxon rank‐sum test. Categorical variables were expressed as frequency (%) and were compared using the chi‐square or Fisher exact test. The Spearman correlation analyses were used to assess the correlations between serum sodium or chloride with interested factors. In addition, univariate and multivariate nonconditional logistic regression models were used to assess the association of serum sodium and chloride with dyskinesia in PD patients. Odd ratios (ORs) and 95% confidence intervals (95% CIs) were used to evaluate the risk of dyskinesia. The covariates included in the multivariable models were age, sex, age at onset of PD, duration of disease, daily levodopa‐equivalent dose (LED), H&Y stage, second part of Unified Parkinson Disease Rating Scale (UPDRS II) and third part of Unified Parkinson Disease Rating Scale (UPDRS III) scores, cigarette smoking, alcohol consumption, coffee consumption, history of hypertension, history of diabetes mellitus, and motor fluctuation. Receiver operating characteristic (ROC) curves were configured to establish cutoff points of serum sodium and chloride that optimally predicted dyskinesia. All *p* values were two‐tailed, and a significance level of .05 was used. Statistical analysis was conducted using SAS statistical software (version 9.2, Cary, North Carolina, USA).

## 3 RESULTS

A total of 102 patients were included in this study. The baseline characteristics are presented in Table 1. Patients with dyskinesia tended to have longer duration of disease, higher daily LED, and H&Y stage, with lower levels of serum sodium than those without dyskinesia. There were no significant differences in serum chloride or other variables between the two groups. Considering the medication effects on serum sodium and chloride concentration, we also collected the medication information related to the renin–angiotensin aldosterone system in PD patients. There were only 3 patients using Angiotensin II receptor blockers(ARBs) in patients without dyskinesia, and was nobody using ARB in patients with dyskinesia. There was no statistical difference between the two group (*p *=* *.400). None of these patients used Angiotensin‐converting Enzyme Inhibitors(ACEI).

**Table 1:** Baseline characteristics of patients with Parkinson's disease

Characteristic^a^	Patients without Dyskinesias (*n *=* *83)	Patients with Dyskinesias (*n *=* *19)	*p*‐value
Age, y	64.6 ± 9.3	66.0 ± 9.0	.555
Male sex	42 (50.6)	9 (47.4)	.799
Age at onset of PD, y	58.9 ± 8.8	54.8 ± 8.6	.076
Duration of disease, y	5.4 ± 4.1	10.4 ± 4.6	<.001
Daily LED, mg/d	400.0 (300.0–501.4)	690.0 (500.0–792.0)	<.001
Hoehn–Yahr stage	2.0 (1.5–2.5)	2.5 (2.0–3.0)	.048
UPDRS II	11.0 (8.0–16.0)	14.0 (10.0–18.0)	.211
UPDRS III	22.0 (17.0–34.0)	26.0 (19.0–43.0)	.197
Cigarette smoking	19 (22.9)	7 (36.8)	.246
Alcohol consumption	16 (19.3)	5 (26.3)	.534
Coffee consumption	5 (6.0)	1 (5.3)	1.000
History of hypertension	18 (21.7)	3 (15.8)	.757
History of diabetes mellitus	6 (7.2)	2 (10.5)	.640
Motor fluctuation	55 (66.3)	15 (79.0)	.283
Serum sodium, mmol/L	142.5 (140.3–145.0)	140.1 (137.1–142.7)	.007
Serum chloride, mmol/L	103.3 (102.1–105.0)	102.8 (95.1–105.8)	.190

PD, Parkinson's disease; LED, levodopa equivalent dose; UPDRS II, second part of Unified Parkinson Disease Rating Scale score; UPDRS III, third part of Unified Parkinson Disease Rating Scale score.

a Continuous variables are expressed as mean ± standard deviation or as median (interquartile range). Categorical variables are expressed as frequency (percent).

The spearman correlation analyses showed that serum sodium inversely correlated with duration of disease (*r* = −.218, *p *=* *.028), and positively correlated with serum chloride levels (*r* = .565, *p *<* *.001; Table 2); in addition, serum chloride was inversely correlated with cigarette smoking (*r* = −.198, *p *=* *.046) and motor fluctuation (*r* = −.220, *p *=* *.027). Univariate logistic regression analysis found that duration of disease, daily LED, serum sodium, and serum chloride were associated with dyskinesia in PD patients (*p *<* *.05 for all; Table 3). After adjusting for age, sex, age at onset of PD, duration of disease, daily LED, H&Y stage, UPDRS II and UPDRS III scores, cigarette smoking, alcohol consumption, coffee consumption, history of hypertension, history of diabetes mellitus, and motor fluctuation, serum sodium, and chloride were still associated with dyskinesia, with corresponding ORs of 0.783 (95% CI, 0.642‐0.955) and 0.796 (95% CI, 0.652‐0.972), respectively (Table 4). The optimal serum sodium and chloride cut points (141.1 mmol/L and 100.9 mmol/L, respectively) were obtained from the ROC curves.

**Table 2:** Spearman correlation coefficients of serum sodium and chloride with other factors among Parkinson's disease patients

Variables	Serum sodium		Serum chloride	
	Coefficient	*p*‐value	Coefficient	*p*‐value
Age	−0.014	.885	0.122	.222
Sex	0.146	.144	0.156	.117
Age at onset of PD	0.151	.130	0.126	.207
Duration of disease	−0.218	.028	−0.080	.422
Daily LED	−0.094	.350	−0.084	.399
Hoehn–Yahr stage	−0.078	.445	0.109	.281
UPDRS II	−0.124	.213	−0.083	.409
UPDRS III	0.026	.799	0.121	.226
Cigarette smoking	−0.004	.970	−0.198	.046
Alcohol consumption	0.035	.730	−0.089	.372
Coffee consumption	0.192	.053	−0.023	.821
History of hypertension	0.006	.951	−0.124	.213
History of diabetes mellitus	−0.058	.565	−0.107	.284
Motor fluctuation	−0.066	.510	−0.220	.027
Serum sodium	–		0.565	<.001
Serum chloride	0.565	<.001	–	

PD, Parkinson's disease; UPDRS II, score of second part of Unified Parkinson Disease Rating Scale; UPDRS III, score of third part of Unified Parkinson Disease Rating Scale.

**Table 3:** Univariate logistic regression analysis of dyskinesia with other factors in Parkinson's disease patients

Variables	Odds ratio (95% confidence interval)	*p*‐value
Age	1.017 (0.962–1.076)	.551
Female sex	1.138 (0.420–3.088)	.799
Age at onset of PD	0.951 (0.898–1.006)	.080
Duration of disease	1.260 (1.116–1.422)	<.001
Daily levodopa‐equivalent dose	1.003 (1.001–1.005)	.001
Hoehn–Yahr stage	1.531 (0.906–2.586)	.111
UPDRS II	1.055 (0.989–1.126)	.104
UPDRS III	1.021 (0.991–1.052)	.164
Cigarette smoking	1.965 (0.678–5.692)	.213
Alcohol consumption	1.496 (0.470–4.760)	.495
Coffee consumption	0.867 (0.095–7.879)	.899
History of hypertension	0.677 (0.178–2.584)	.568
History of diabetes mellitus	1.511 (0.280–8.138)	.631
Motor fluctuation	1.909 (0.579–6.295)	.288
Serum sodium	0.808 (0.702–0.931)	.003
Serum chloride	0.867 (0.769–0.978)	.020

PD, Parkinson's disease; UPDRS II, score of second part of Unified Parkinson Disease Rating Scale; UPDRS III, score of third part of Unified Parkinson Disease Rating Scale.

**Table 4:** Multivariate logistic regression analysis of serum sodium and chloride and dyskinesia in Parkinson's disease patients

Variables	Odds ratio (95% confidence interval)	*p*‐value
Serum sodium and dyskinesia		
Serum sodium	0.783 (0.642–0.955)	.016
Daily Levodopa‐equivalent dose	1.003 (1.000–1.006)	.049
Serum chloride and dyskinesia		
Serum chloride	0.796 (0.652–0.972)	.025
Daily levodopa‐equivalent dose	1.003 (1.000–1.006)	.049

Adjusted for age, sex, age at onset of PD, duration of disease, levodopa‐equivalent daily dose, Hoehn–Yahr stage, UPDRS II, UPDRS III, cigarette smoking, alcohol consumption, and coffee consumption.

## 4 DISCUSSION

Our study demonstrated that serum sodium and chloride are associated with dyskinesia in patients with PD. The correlation of serum sodium and chloride with dyskinesia remained significant, even with adjustment for several potential confounders. We have also demonstrated that patients with lower level of serum sodium and chloride are more likely to have dyskinesia.

Because of the impact of dyskinesia on treatment of PD, and the negative effects of dyskinesia on patients’ and their caregivers’ quality of life, strategies to prevent dyskinesia are needed, as is identification of the risk factors for dyskinesia. Several risk factors were found to be associated with the development of dyskinesia, including age of onset of PD, disease duration, female sex, and the level of exposure to L‐(Fahn, 2006; Guigoni & Bezard, 2009; Mann et al., 2012; Schrag & Quinn, 2000; Silverdale et al., 2010). Our study showed patients with dyskinesia tended to have longer duration of disease, higher daily LED, and H&Y stage, with lower levels of serum sodium than those without dyskinesia. Multivariate logistic regression analysis suggested that serum sodium and chloride were inversely associated with dyskinesia in PD patients.

Sodium is a vital component of the human body. In neurons, the concentration of sodium ions outside is roughly ninefold larger than inside. The constant gradient of concentrations across the membrane maintained by sodium‐potassium pump plays an important role in physiological osmosis, transferring of electric potential and calcium regulation, cell volume control, glucose transport by sodium channels, the electric potential of membranes, pH regulation, and neurotransmission (Ha, Jeong, Kim, & Churchill, 2016). Nav 1.6 sodium channels in the globus pallidus which play an important role in the regulation of speed and fast spiking in neuronal systems (Mercer, Chan, Tkatch, Held, & Surmeier, 2007). Selective inhibition of striatal fast‐spiking interneurons causes dyskinesia (Gittis et al., 2011). Low extracellular sodium causes detrimental effects in neuronal cells. Hyponatremia could lead to osmotic demyelination syndrome (ODS). Osmotic brain injury due to hyponatremia triggers apoptosis in astrocytes followed by a loss of trophic communication between astrocytes and oligodendrocytes, secondary inflammation, microglial activation, and finally demyelination (Gankam Kengne et al., 2011). Chronically and abnormally activated astrocytes lead to an aberrant neuron‐glia communication, which affect synaptic activity and neuroplasticity contributing to the development of LID (Carta et al., 2017). Osmotic edema could also increase neuronal excitability through activation of NMDA receptors (Lauderdale et al., 2015). NMDA receptor antagonists have been shown to exert a beneficial effect in blocking the development of dyskinesia in experimental models of LID (Bastide et al., 2015).

Chloride is the most abundant electrolyte in serum after sodium, and is also very important in the regulation of body fluids, electrolyte balance, the preservation of electrical neutrality, and acid‐base status (Powers, 1999). In the adult brain, chloride is 10–20‐fold lower than the extracellular concentration, leading to a large driving force for inward chloride diffusion. Chloride homeostasis has a pivotal role in controlling neuronal excitability in the adult brain and during development. Chloride fluxes indicate the activation of the ionotropic GABA currents, which are the main mediator of synaptic inhibition in the postnatal cortex. Intraneuronal chloride concentration regulation impacts on both cell volume homeostasis and chloride‐permeable GABAA receptor‐dependent membrane excitability (Glykys et al., 2017). Effective fast inhibition which is crucial for proper neural coding in the central nervous system is mediated primarily by Cl currents through GABAA and glycine‐gated receptors/channels (Doyon et al., 2016). Intracellular chloride concentration is low in neurons, and the reversal potential for chloride currents is close to the resting membrane potential. Thus, even small changes in intracellular chloride concentration can significantly affect the strength and even polarity of GABA/glycine‐mediated transmission. Modulating chloride gradients even comes out as a mechanism by which microglia can control neuronal excitability (De Koninck, 2007). Increased death of dopaminergic neurons due to hypochlorite‐dependent conversion into a cytotoxic redox‐cycling product may be a factor in the pathogenesis of PD(Mehta et al., 2016). In addition, calcium homeostasis which is closely related to serum sodium and chloride is found to be dysregulated in PD patients with LID (Blandini et al., 2009).

All these findings suggest the possibility of a inverse association between serum sodium and chloride and dyskinesia in PD patients as what we have found in our study.

In summary, we have demonstrated that serum sodium and chloride are inversely associated with dyskinesia in PD patients. Considering the low salt diet of many elderly individuals and most of the PD patients, and diuretics use in PD patients with hypertension, which always potentially lead to decrease the level of serum sodium and chloride, we should pay more attention to avoid the low serum sodium and chloride in PD patients, especially those with relatively large amount of daily LED and longer disease duration who are at high risk for dyskinesia.

### 4.1 Limitations

Our study has several limitations. First, our study sample is relatively small and the patients were just from the Second Affiliated Hospital of Soochow University, so the study findings may be limited by the relatively small population from a single center. Second, this is a retrospective study, and patients were enrolled in this study only if they had electrolyte testing which may also contribute to selection bias. Moreover, we only did one single measurement of fasting electrolytes which may not be an accurate estimation of serum sodium and chloride. Further study with larger numbers of subjects from different centers and different serum sodium and chloride levels (low, normal, and high) is needed.

## CONFLICTS OF INTEREST

The authors declare that they have no conflict of interest.
